# The effects of patients initiated aggression on Chinese medical students’ career planning

**DOI:** 10.1186/s12913-017-2810-2

**Published:** 2017-12-28

**Authors:** Zhonghui Xie, Jing Li, Yuhua Chen, Kaijun Cui

**Affiliations:** 1 Institute of Clinical Medicine, West China Medical School of Sichuan University, Chengdu, China; 2 Department of Endocrinology, West China Hospital of Sichuan University, Chengdu, China; 3 Department of Cardiac Surgery, West China Hospital of Sichuan University, Chengdu, China; 4 Department of Cardiology, West China Hospital of Sichuan University, Chengdu, China

**Keywords:** Patient initiated aggression, Medical students, Effects, Career planning, Quality of life

## Abstract

**Background:**

Patient initiated aggression is common among Chinese health-care workers, reaching over 10,000 incidents annually (Jinyang web. http://6d.dxy.cn/article/55497. 2013), and the tense doctor-patient relationship generates stress among medical students. Because of the paucity of data (few surveys pay attention to the effects of violence perpetrated by patients on medical students), this study aimed to characterize patient initiated aggression against medical students.

**Methods:**

In this cross-sectional survey conducted at a medical school in West China in 2015, 157 medical students completed a self-administered questionnaire and the Short Form-36, which assesses quality of life. The associations between patient initiated aggression exposure and medical students’ career planning or quality of life were assessed using a chi-square test.

**Results:**

Of the 157 medical students, 48 (30.6%) reported having suffered patient initiated aggression at least once during the previous year in the form of mental abuse (20.4%), offensive threat (14.6%), physical violence (8.3%), sexual harassment (verbal: 8.3% or physical: 1.6%), and extreme violence (physical violence leading to surgical treatment or hospitalization) (0.6%). Insufficient communication was the primary reason cited (27.2%). Emotional attack (mental abuse and offensive threat) occurrence differed among age groups (χ^2^ = 9.786, *P* = 0.020) and was ubiquitous among those aged >30 years old. Women were more likely than men to suffer physical violence (χ^2^ = 6.796, *P* = 0.009). Patient initiated aggression was not significantly associated with medical students’ career planning or quality of life.

**Conclusions:**

In this study, patient initiated aggression, albeit common, as in the rest of China, did not appear to be associated with medical students’ career planning or quality of life. However, the characteristics described can inform policymaking and the design of programs to minimize patient initiated aggression occurrence.

## Background

Medical providers should be respected staff members. However, in China, their personal safety is threatened because of high treatment costs, strained medical resources, and an imperfect health-care system (unreasonable health investment and medical insurance system) caused by medical marketization. Patients are often unable to address these health-care system defects, so they are prone to vent their frustration on medical staff. The number of incidents of violence perpetrated on health-care workers in Chinese hospitals by patients or their relatives has reached over 10,000 annually [1], and the tendency is increasing. From October 21, 2013 to October 4, 2016, the media covered at least 35 patient initiated aggression incidents, 19 of which resulted in extreme injury or death, 15 in no serious consequences, and at least 11 in disruption of the hospital’s basic functioning [2–7]. Several studies have evaluated the prevalence of patient initiated aggression in developing countries such as China, Pakistan, Palestine, and Turkey. Previous studies indicated that the prevalence of patient initiated aggression among medical staff members was 76.02% in China, 72.5% in Pakistan, 76.1% in Palestine, and 44.7% in Turkey [7–10]. The main reason underlying the deteriorating relationship between patients and doctors is the relative scarcity of medical resources in mainland China [11] and a lack of understanding of the problem by policymakers. The Chinese Ministry of Health and the Ministry of Public Security put forth a policy of adding security guards in hospitals. However, this policy failed to address the fundamental issue of protection of health-care professionals while perhaps even further intensifying doctor–patient conflicts [4]. Chinese physicians thus remain under a large amount of stress [12], as reflected by the common occurrence of anxiety and depressive symptoms [13, 14]. Patient initiated aggression likely interferes with diagnosis and treatment [15–19], is hazardous to the physical and mental health of medical staff [13, 20–22], and adversely affects medical student recruitment [1]. Increasingly fewer senior high school students are willing to register for medical school, leading to extreme lack of physician faculty. Most physicians do not want their children to engage in medical work. Although medical students also suffer from stress caused by tense doctor–patient relationships, studies are scarce which characterize the effects of patient initiated aggression on medical students’ quality of life and career planning, which is the subject of this survey-based study in a West China medical school.

## Methods

### Participants and sampling

The study was a cross-sectional survey conducted in 2015 in the West China Clinical Medicine School. Overall, there were 180 eligible participants. After being informed about the purpose of the study and providing verbal consent, 180 randomly recruited medical students completed a self-administered questionnaire along with the Short Form-36 (SF-36) scale testing quality of life. 23 (12.8%) refused to participate, and finally 157 were qualified for this study, with an overall response rate of 87.2%.

### Measurement

The questionnaire contained five sections covering demographic characteristically, characteristics and causes of violence experienced during the previous 12 months, and the effects of such violence on the medical students including their quality of life. Demographic characteristics included age, gender, marital status, educational level, work experience in health sectors, mean daily hours at work, weekly night shift, weekly clinic service visits, and monthly discharges. Characteristics of violence during the previous 12 months included extreme violence (physical violence led to surgical treatment or hospitalization), physical violence (students suffered physical damage), mental abuse (patients used language to insult students), offensive threats (patients verbally expressed the intention for physical violence), verbal sexual harassment (patients verbally flirted with students), and physical sexual harassment (patients physically flirted with students). The three dimensions of patient initiated aggression were emotional attack (EA, mental abuse and offensive threat), sexual harassment (SH, verbal and physical sexual harassment), and physical violence (PV, extreme violence and personal attack). Items investigating the occupational plan were added to the patient initiated aggression scale. Causes of violence included insufficient communication (i.e., the victim was a poor communicator; medical students are usually less experienced than physicians, leading to insufficient doctor–patient communication, so the patient may not make the most informed decision about their care, which is likely to cause conflicts), lack of trust between patients and physicians, low-level personal quality (meaning a person who is rude and prone to address problems by violence and does not respect people; put another way, this is a problem of the perpetrator lacking a humanistic quality), staff attitudes, unmet expectations of patients/families, waiting time, out-of-pocket fees, patient’s death, emergency circumstances, lack of medicines or needed services, and others. These were fixed choices. We read some papers containing questionnaire designs relating to our study (*Health-care personnel and patient initiated aggression in the emergency departments of a volatile metropolis: results from Karachi, Pakistan* [8]; *Workplace towards workers in the emergency departments of Palestinian hospitals: a cross-sectional study* [9]; and *Chinese doctors are under threat* [12], which we have cited in our paper). Effects of violence on medical students included pursuing a medical career, choosing a non-medical career, and giving up a medical career. Medical students’ quality of life was measured using the SF-36 scale, comprehensively summarizing respondents’ survival quality based on eight aspects: physical functioning, role physical, bodily pain, general health perceptions, vitality, social role functioning, role emotional, and mental health. The SF-36 scale had high consistency with a Cronbach’s α coefficient greater than 0.7 (except for social role functioning and vitality).

### Statistical analysis

All statistical analyses were performed using SPSS software (version 19.0). Descriptive statistics were used for demographic characteristics, violence characteristics, causes, and effects. A chi-square test was used to examine possible correlations between patient initiated aggression and medical students’ career planning or quality of life. A two-tailed *P* < 0.05 was accepted as statistically significant in all analyses.

## Results

### Medical student characteristics

The survey response rate for the questionnaire and SF-36 form was 87.2%. Participants predominantly were 20–24 years old (66.3%), female (56.1%), and single (96.2%) and worked 6–8 h daily (54.8%) with almost all having night shifts (96.2%) (Table 1).

**Table 1:** Demographic characteristics of the 157 medical students

Characteristics		n (*N* = 157)	%
Age	<19 years	3	1.9
	20–24 years	104	66.3
	25–29 years	47	29.9
	>30 years	3	1.9
Gender	Male	67	42.7
	Female	88	56.1
Marital status	Divorced	2	1.3
	Not Married	151	96.2
	Married	4	2.5
Educational level	Bachelor degree	120	76.4
	Master degree	14	8.9
	Doctor degree	23	14.6
Work experience in the health sector	<6 months	52	33.1
	6–12 months	53	33.8
	12–24 months	44	28.0
	24–36 months	3	1.9
	>36 months	5	3.2
Hours worked daily	<6 h	9	5.7
	6–8 h	86	54.8
	8–10 h	41	26.1
	10–12 h	18	11
	>12 h	3	1.9
Night shifts/week	<1	34	21.7
	1	71	45.2
	>1	46	29.3
	0	6	3.8
Clinic service visits/week	<5	99	63.1
	5–9	19	12.1
	10–14	7	4.5
	15–19	6	3.8
	>20	26	16.6
Discharges/month	<5	49	31.2
	5–9	35	22.3
	10–14	24	15.3
	15–19	15	12.1
	>20	30	19.1

### Prevalence of violence against medical students

Of the 157 medical students surveyed, 48 (30.6%) reported having suffered patient initiated aggression at least once during the previous year in the form of mental abuse (32, 20.4%), offensive threats (23, 14.6%), PV (13, 8.3%), SH (verbal: 13, 8.3% or physical:3, 1.6%), and extreme violence (PV leading to surgical treatment or hospitalization) (1, 0.6%). Among reasons for violence, insufficient communication was the most common (27.2%) followed by lack of trust between patients and physicians (25.7%), patient’s low-level personal quality (13.2%), staff member’s attitudes (10.6%), unmet expectations of patients/families (6.9%), waiting time (5.6%), out-of-pocket fees (4.3%), and patient’s death (3.5%).

The three dimensions of patient initiated aggression were distributed as follows: EA (41 times, 26.1%), SH (15 times, 9.6%), and PV (13 times, 8.3%). In analyses by gender, age, marital status, educational level, and daily working hours, EA occurrence differed among age groups (χ2 = 9.786, *P* = 0.020), with participants aged >30 years more likely to suffer EA than participants aged 20–24 years (χ2 = 7.567, *P* = 0.006) and participants aged 25–29 years (χ2 = 10.106, *P* = 0.001). Women were more likely exposed to PV (χ2 = 6.796, *P* = 0.009) (Table 2). Patient initiated aggression appeared not to impact medical students’ career planning over the following three years; most medical students (89.2%) were inclined to continue their medical careers. Only 17 students decided to choose non-medical careers (12.5%) or to give up their medical career (2.1%) (Table 3).

**Table 2:** Demographic characteristics of medical students by violence dimension

	N.	All violence		PV		EA		SH	
		n.	Rate (%)	n.	Rate (%)	n.	Rate (%)	n.	Rate (%)
Gender									
Male	67	22	32.8	3	3.3	21	23.3	9	10.0
Female	88	26	28.9	10	14.9	20	29.9	6	9.0
χ2		0.282		6.796		0.846		0.049	
*P* value		0.595		0.009		0.358		0.826	
Age									
<19 years	3	1	33.3	1	33.3	1	33.3	0	0.0
20–24 years	104	32	30.8	8	7.7	28	26.9	11	10.6
25–29 years	47	12	25.5	3	6.4	9	19.1	3	6.4
>30 years	3	3	100.0	1	33.3	3	100.0	1	33.3
χ2		7.388		5.229		9.786		2.953	
*P* value		0.061		0.156		0.020		0.399	
Educational level									
Bachelor degree	120	34	28.3	7	5.8	28	23.3	11	9.2
Master degree	14	6	42.9	2	14.3	5	35.7	1	7.2
Doctor degree	23	8	34.8	4	17.4	8	34.8	3	13.0
χ2		1.417		4.125		2.045		0.439	
*P* value		0.479		0.127		0.360		0.803	
Marital status									
Married	151	45	29.8	12	7.9	38	25.2	14	9.3
Not Married	4	2	50.0	0	0.0	2	50.0	1	25.0
Divorced	2	1	50.0	1	50.0	1	50.0	0	0.0
χ2		1.109		4.967		1.845		1.330	
*P* value		0.574		0.083		0.398		0.514	
Mean working hours									
<6 h	9	0	0.0	0	0.0	0	0.0	0	0.0
6–8 h	86	25	29.1	4	4.7	20	23.3	6	7.0
8–10 h	41	17	41.5	5	12.2	15	36.6	7	17.1
10–12 h	18	4	22.2	3	16.7	4	22.2	1	5.6
>12 h	3	2	66.7	1	33.3	2	66.7	1	33.3
χ2		8.778		7.278		8.573		6.590	
*P* value		0.067		0.122		0.073		0.159

**Table 3:** effects of medical violence on medical students’ career planning

(Chi-square = 1.655,*P* = 0.437)						
Career planning	Total		medical students not suffering violence		medical students who suffered violence	
	N.	Rate	N.	Rate	N.	Rate
Pursuing medical career	140	89.2	99	90.8	41	85.4
Choosing non-medical career	13	8.3	7	6.4	6	12.5
Giving up medical career	4	2.5	3	2.8	1	2.1
Total	157	100.0	109	100.0	48	100.0
PV (Chi-square = 0.383,*P* = 0.826)						
Career planning	Total		medical students not suffering PV		medical students who suffered PV	
	N.	Rate	N.	Rate	N.	Rate
Pursuing medical career	140	89.2	128	88.9	12	92.7
Choosing non-medical career	13	8.3	12	8.3	1	7.7
Giving up medical career	4	2.5	4	2.8	0	0.0
Total	157	100.0	144	100.0	13	100.0
EA (Chi-square = 2.951,*P* = 0.229)						
Career planning	Total		medical students not suffered EA		medical students suffered EA	
	N.	Rate	N.	Rate	N.	Rate
Pursuing medical career	140	89.2	106	91.4	34	82.9
Choosing non-medical career	13	8.3	7	6.0	6	14.6
Giving up medical career	4	2.5	3	2.6	1	2.4
Total	157	100.0	116	100.0	41	100.0
SH (Chi-square = 0.946,*P* = 0.623)						
Career planning	Total		medical students not suffered SH		medical students suffered SH	
	N.	Rate	N.	Rate	N.	Rate
Pursuing medical career	140	89.2	127	89.4	13	86.7
Choosing non-medical career	13	8.3	11	7.7	2	13.3
Giving up medical career	4	2.5	4	2.8	0	0.0
Total	157	100.0	142	100.0	15	100.0

### Relationship between patient initiated aggression and medical students’ quality of life

The SF-36 scale scores among medical students by frequency of patient initiated aggression showed that most medical students’ patient initiated aggression score were concentrated in the zero frequency group (69.4%), while there were 48 medical students in the intermediate frequency group (30.6%). Comparing the SF-36 scores in terms of the difference in patient initiated aggression frequency, the score of the low–intermediate frequency group was lower than in the zero frequency group. The students’ quality of life, including physical functioning, role physical, bodily pain, vitality, social role functioning, role emotional, and mental health physical functioning, in the intermediate frequency group was moderately affected by patient initiated aggression.

From the data in Table 4, it is apparent that the scores of the eight dimensions of the SF-36 scale do not have strong correlations with patient initiated aggression frequencies. Additionally, correlation coefficients are not significantly different, which renders it unfeasible to assess the correlation between medical students’ quality of life and patient initiated aggression. In conclusion, patient initiated aggression did not affect the medical students’ quality of life.

**Table 4:** Correlation among 8 dimensions of SF-36 and medical violence score

		SF-36 total score	PF	RP	BP	GH	VT	SF	RE	MH
Total frequency	Pearson correlation coefficient	−0.068	−.021	−.073	−.055	.005	.025	−.088	−.073	−.111
	P value	0.398	.793	.368	.495	.946	.759	.275	.368	.166
	N.	155	157	156	156	157	157	157	156	157
PV frequency	Pearson correlation coefficient	−.061	−.065	−.046	−.091	.016	.078	−.104	−.046	−.128
	P value	.453	.421	.565	.261	.846	.332	.196	.565	.111
	N.	155	157	156	156	157	157	157	156	157
EA frequency	Pearson correlation coefficient	−.091	.007	−.112	.005	.011	−.013	−.076	−.112	−.163*
	P value	.261	.929	.164	.953	.893	.870	.345	.164	.041
	N.	155	157	156	156	157	157	157	156	157
SH frequency	Pearson correlation coefficient	.052	−.023	.022	−.093	−.014	.034	−.038	.022	.061
	P value	.519	.771	.787	.246	.859	.673	.640	.787	.447
	N.	155	157	156	156	157	157	157	156	157

Pearson correlation coeffecient with statistical significance is marked in *

## Discussion

An ENT (ear-nose-throat) doctor was killed by a patient who was unsatisfied with operation effectiveness with knife and hammer. While an ENT doctor working in outpatient department, a patient smashed his head with a 50-cm long pipe. From 2013 to 2016, media covered over 35 patient initiated aggression incidents with an upward tendency. Doctors’ work environment is stressed. Are medical students willing to engage in this high risk job?

Because few surveys pay attention to the effects of violence perpetrated by patients on medical students, we compared our data with those of a survey about patient initiated aggression against physicians (percentages below were incident rates). This survey among medical students in a West China program revealed a frequency of patient initiated aggression exposure of 30.6%, which is lower than those reported in previous studies for medical staff members in China, namely 76.02%, 50%, and 82.4% [7, 13, 14]. This might be explained by the greater contact time with patients of physicians relative to medical students. Among violence dimensions, EA (35%) was more prevalent than PV (8.9%), as had been reported in studies in Pakistan (EA 72.6%, PV 16.5%) [8], Ipswich (EA 47.91%, PV 20.08%) [21], and Turkey (EA 43.2%, PV 6.8%) [10]. Additionally, in the present study, EA occurrence differed by age, affecting all individuals older than 30 years old. This finding is similar to the results of a Palestinian study [9] in which individuals over 30 were more likely to suffer non-physical violence.

Regarding the reasons for patient initiated aggression, insufficient communication (27.2%) was the primary reason in the present study, which was consistent with a South China study (93.0%) [22]. Waiting time was the most common cause in the Palestinian study (47.5%) [9].

Patient initiated aggression did not influence medical students’ quality of life in the present study, in contrast to a previous study demonstrating that patient initiated aggression resulted in anxiety and depression symptoms among Chinese physicians [13]. The latter difference in results might be explained by the fact that medical students work under the supervision of medical professors and experienced physicians, which may lower stress levels.

Additionally, patient initiated aggression did not change the medical students’ career planning. There are many reasons contributing to this. Primarily, China’s criminal law has been reformed. If the patient initiated aggression is serious enough to constitute a crime, the assaulters will face sanctions under China’s criminal law. Therefore, medical students might think the new criminal law will improve the present situation before they become professional physicians and suffer more patient initiated aggression. Then, a substantial salary tempts students to engage in medical work. Further, medical students are usually chosen from among those excelling in the national college entrance examination, and they might be less willing to forego the hard-won opportunity to pursue their dreams of a medical career.

Methods should be undertaken to prevent violence. First, measures to improve safety require significant health-care system changes to tackle the roots of conflict [23, 24]. Second, social media should also play a more constructive role in conflict mitigation by avoiding sensationalist coverage of violent events, which might have contributed to worsening doctor-patient conflicts [7]. Third, automatic door locks and some safety measures have proved useful in emergency situations as reported by Morken [25], Touzet [26], Feldmann [27], and Mills [28]. Fourth, teaching health-care workers to address patient expectation mismatch was vital to conflict prevention [29, 30]. A zero-tolerance attitude toward patient initiated aggression should prevail [31].

## Conclusions

Along with revealing a lower prevalence of patient initiated aggression in medical students as compared to that reported in studies of more experienced medical staff in hospitals in China, the present study failed to document an association between patient initiated aggression and medical students’ career planning or quality of life. This may be due to the fact that the majority (66.9%) of respondents’ work experience was less than 12 months. However, the characteristics of patient initiated aggression described for medical students inform policymaking to minimize possible medical staff turnover by reforming the health-care system and providing students with better job prospects. It is not too late to focus on remediating the situation so that medical students will not have second thoughts about their career choice; a shortage of physicians would only aggravate looming health-care crises.

### Limitations

There are several limitations in our study. First, a cross-sectional study cannot determine causality between patient initiated aggression and changes in medical students’ career planning or quality of life. Second, the self-reported nature of study data might introduce bias and errors. Finally, study participants practiced in a large hospital of a large city, which does not allow assessment of the situation in rural areas or small hospitals. The post hoc power is lower than 0.8, so this paper’s negative results is less credible. Large sample and longitudinal studies are warranted to confirm the findings and the issues of resources as a risk of violence.

## Abbreviations

EA: Emotional attack
ENT: Ear-nose-throat
PV: Physical violence
SF-36: Short Form-36
SH: Sexual harassment
SPSS: Statistical package for social sciences
